# Sustainable energy transitions in sports tourism: bridging infrastructure investment and event legacy planning

**DOI:** 10.3389/fspor.2026.1794795

**Published:** 2026-03-12

**Authors:** Hamida Toyirova, Parviz Toyirov

**Affiliations:** 1 Department of Tourism and Hotel Management, Faculty of Economics and Tourism, Bukhara State University, Bukhara, Uzbekistan; 2 Department of Project Management, Graduate School of Business and Entrepreneurship, Tashkent, Uzbekistan

**Keywords:** affective atmospheres, climate action, eco-anxiety, embodiment, emotion, event legacy, green infrastructure, place attachment

## Abstract

How do people come to care about the environmental footprint of the places where they cheer, compete, and celebrate? This conceptual analysis argues that the answer lies not in information alone but in what bodies feel. Sustainable energy transitions in sports tourism venues—geothermal heating that steadies the air, solar arrays that reshape rooflines, natural ventilation that reconnects indoor arenas with the outdoors—alter the sensory fabric of sporting spaces in ways that generate distinctive emotional responses: comfort, pride, hope, and sometimes moral dissonance. Drawing on phenomenological theories of embodiment, the sociology of emotion in sport, and the concept of affective atmospheres, we develop the Embodied Sustainable Sports Experience (ESSE) Framework. The ESSE Framework maps three interconnected layers—somatic encounter, affective response, and identity-behavior translation—through which bodily experiences of green infrastructure may catalyze engagement with climate action. We illustrate the framework through conceptual application to mega-event legacies and national energy transitions in Central Asia, arguing that emerging sports tourism destinations hold particular promise for embedding sustainability into the lived, felt texture of sporting life. This analysis contributes to the interdisciplinary dialogue on how sport—as a uniquely embodied and emotionally charged domain of human experience—can move people toward environmental consciousness and action.

## 1 Introduction

Stand in a crowd of forty thousand on a midsummer evening and you will understand, in the most bodily way possible, that sport is not merely watched—it is felt. The press of shoulders, the vibration of a collective roar travelling through your ribcage, the sweat that comes from heat and from anticipation: these sensations bind spectators to place and to each other with a force that no broadcast can replicate. It is precisely this embodied intensity that makes sports tourism venues such potent—yet overlooked—sites for shaping how people relate to the environments around them, including the energy systems that keep those environments habitable.

The global sports tourism industry has become a formidable economic and cultural force, generating employment, infrastructure development, and cross-cultural exchange across host destinations worldwide (8, 9). At the same time, the imperative for sustainable energy management has intensified under the Paris Agreement and growing stakeholder expectations for environmentally responsible operations. These twin forces have produced a growing body of scholarship on green sports infrastructure—yet the conversation has remained overwhelmingly technical. Researchers ask how many solar panels a stadium roof can support, what the payback period for geothermal heating might be, and how much carbon can be offset through renewable procurement (10, 11).

These are important questions. But they miss something fundamental about what happens inside a sports venue. They treat the people who inhabit these spaces—athletes whose lungs draw in the air, spectators whose skin registers the temperature, communities whose daily lives unfold in the shadow of the arena—as passive beneficiaries or abstract economic units rather than as sensing, feeling bodies whose experiences of place profoundly shape their relationship with environmental issues. As Vannini et al. (12) remind us, places are not neutral containers; they are constituted through the sensory, emotional, and kinesthetic engagements of the people who move through them.

This conceptual analysis addresses that gap. We ask: how do embodied and emotional experiences within sustainably transformed sports venues shape engagement with climate action? In posing this question, we respond to calls within sports studies for greater attention to the lived, felt dimensions of sporting environments (2, 13) and to the emerging recognition that environmental attitudes are rooted not only in cognition but in bodily sensation and affective response (14, 15).

Our argument proceeds in four stages. First, we lay the theoretical groundwork by drawing on phenomenological approaches to embodiment, the sociology of emotions in sport, and the concept of affective atmospheres. Second, we examine how sustainable energy infrastructure creates distinctive somatic and emotional experiences across four sensory dimensions. Third, we propose the Embodied Sustainable Sports Experience (ESSE) Framework, mapping pathways from physical sensation through emotional response to climate-conscious identity and behavior. Fourth, we consider implications for emerging sports tourism destinations, particularly in Central Asia, where the convergence of rapid tourism development and energy transition offers distinctive opportunities to weave sustainability into the very feel of sporting life.

## 2 Theoretical foundations: bodies, feelings, and sporting environments

### 2.1 Embodiment and the phenomenology of sport

The concept of embodiment, developed in the phenomenological tradition from Merleau-Ponty (1) onward, insists that human beings do not merely have bodies—they are bodies. Perception, understanding, and meaning-making are fundamentally corporeal processes. We do not first think about the world and then act in it; we come to know the world through our bodily engagement with it. In sports studies, this insight has been developed by scholars who demonstrate that sporting knowledge is “in the muscles” (16), that athletic skill is a form of embodied intelligence, and that the sporting environment is experienced through a constant, pre-reflective dialogue between body and surroundings (2, 13).

This phenomenological lens has profound implications for how we understand the relationship between sports venues and the people who use them. A stadium is not simply a container for athletic performance; it is an environment that is breathed, heard, felt on the skin, and navigated through movement. When the energy systems that power a venue change—when fossil fuel heating gives way to geothermal, when diesel generators are replaced by solar arrays, when ventilation is redesigned for natural airflow—these changes alter the somatic texture of the space itself. Temperature, air quality, humidity, noise, light: every one of these is simultaneously a technical parameter and a lived, bodily experience.

Leder's (17) concept of bodily “dys-appearance”—the way the body comes to conscious attention when something feels wrong, like a headache or stifling heat—is instructive here. In a poorly ventilated, overheated stadium powered by aging fossil fuel infrastructure, spectators and athletes experience bodily discomfort that, while not always articulated, shapes their affective relationship with the venue. Conversely, a well-designed, sustainably powered facility may produce what we call “eu-appearance”: a sense of bodily ease and environmental harmony that, precisely because it feels right, often passes below the threshold of conscious awareness. One of the central challenges for climate communication, as we will argue, lies in making this felt difference visible and meaningful.

### 2.2 Emotions, sport, and environmental concern

Sport is among the most emotionally intense domains of human experience. The elation of victory, the anguish of defeat, the collective effervescence of shared spectatorship—these affective states are not incidental to sport but constitutive of it (18). Sociological and psychological research increasingly recognizes that emotions in sport are not merely individual psychological states but are socially produced, culturally shaped, and spatially situated (3, 19).

The intersection of sport-related emotions with environmental concern is a comparatively new field of inquiry, but several productive threads have emerged. First, the concept of place attachment—the emotional bond between people and meaningful locations (20)—suggests that the deep affective connections spectators and athletes form with sporting venues may extend to concern for the environmental quality of those places. People who love a place tend to care about what happens to it, including its ecological footprint. Second, the growing literature on eco-anxiety and climate emotions (21, 22) suggests that awareness of environmental degradation produces genuine emotional distress that can either motivate action or lead to avoidance and disengagement. Sports venues, as sites of heightened emotional arousal, may amplify these dynamics: the cognitive dissonance of celebrating human achievement inside an environmentally destructive facility, or conversely the alignment and hope experienced in a venue that visibly embodies sustainability values.

Third, Durkheim's concept of collective effervescence, updated for contemporary sports contexts by Cottingham (4), points to the way shared emotional experiences in stadiums create powerful feelings of social solidarity and shared identity. We propose that when these collective emotional experiences occur in sustainably designed environments, they have the potential to forge connections between sporting identity and environmental identity—to make “being green” feel like part of “being us.”

### 2.3 Affective atmospheres and the material environment

Anderson's (5) concept of affective atmospheres provides a crucial bridge between embodiment theory and the material reality of sustainable sports infrastructure. Atmospheres, in this formulation, are neither purely subjective feelings nor purely objective properties of environments; they emerge from the interaction between bodies and their surroundings. A venue's atmosphere is co-produced by its architecture, its energy systems, its sensory qualities, and the embodied responses of the people within it.

This concept is especially relevant because sustainable energy transitions do not merely change the technical specifications of a building—they alter its atmosphere. Natural ventilation creates different airflow patterns and sounds than mechanical systems. Solar-powered LED lighting produces different color temperatures than conventional fixtures. Green roofs and living walls introduce organic textures and even alter olfactory environments. These atmospheric shifts are registered by bodies before they are processed by minds, creating pre-reflective impressions that shape affective orientation toward the space.

Böhme's (23) related work on aesthetic atmospheres suggests that designed environments can intentionally cultivate particular affective tonalities. Applied to sports tourism, this means that sustainable venue design is not merely an engineering challenge but an exercise in emotional architecture—creating spaces that feel right in ways that align with environmental values and promote climate-conscious sensibilities.

## 3 The embodied encounter with green sports infrastructure

How, concretely, does sustainable energy infrastructure alter what it feels like to be in a sports venue? In this section we examine four sensory-somatic dimensions through which green infrastructure reshapes the bodily experience of sporting spaces: thermal sensation, respiratory experience, acoustic perception, and visual-material encounter.

### 3.1 Thermal comfort and the feeling of sustainability

Temperature is perhaps the most immediately felt quality of any built environment, and it matters enormously in sport. Athletes have long recognized that thermal conditions profoundly affect both performance and wellbeing (24), and spectators make unconscious judgments about a venue from the moment they step inside based on whether it feels comfortable or oppressive. Geothermal heating and cooling, solar-thermal installations, and passive design strategies produce thermal environments that differ qualitatively from those generated by conventional HVAC systems.

We suggest that the steady, even warmth provided by geothermal systems—in contrast to the cycling on-off patterns of conventional heating—creates a distinctive thermal atmosphere that bodies register as comfort, stability, and care. Similarly, natural ventilation strategies that allow fresh air circulation produce thermal experiences connecting indoor sporting spaces with the outdoor environment, potentially reinforcing awareness of weather, season, and climate. These are not trivial sensory details; they constitute the background conditions within which sporting emotions are experienced and amplified.

### 3.2 Breathing, air quality, and literal incorporation

The quality of air in a sports venue is literally vital—especially for athletes whose respiratory demands are elevated by exertion. The transition from fossil fuel-dependent energy systems to renewable alternatives reduces local air pollution, altering the respiratory experience of everyone inside. While this change is most pronounced where venues previously relied on diesel generators or coal-fired district heating, even in advanced-economy settings the shift to cleaner energy sources can produce measurable improvements in indoor air quality (11).

From an embodiment perspective, breathing is both the most automatic and the most intimate form of environmental engagement. Every breath draws the environment into the body, making the quality of that environment a matter of literal incorporation. When athletes and spectators breathe cleaner air in a sustainably powered venue, they are, in a very direct sense, taking sustainability into their bodies. This embodied encounter with environmental quality has the potential to make abstract concepts like “clean energy” tangible and personally meaningful in ways that information campaigns alone cannot achieve.

### 3.3 Soundscapes and acoustic experience

Sports venues are acoustic environments of extraordinary intensity—the roar of crowds, the sounds of play, the announcements and music all contribute to the emotional atmosphere that makes live sport compelling. Sustainable energy transitions alter venue soundscapes in subtle but consequential ways. The elimination of diesel generators removes low-frequency mechanical drone; natural ventilation changes the acoustic profile of enclosed spaces; and the absence of cycling air-conditioning compressors can create quieter intervals that allow the organic sounds of sport to emerge more clearly.

Schafer's (25) foundational work on soundscapes reminds us that acoustic environments are not merely backgrounds to human activity but active constituents of experience. The sonic character of a sustainably designed venue contributes to its affective atmosphere, potentially creating acoustic spaces that feel more natural, more connected to the outdoor environment, and more conducive to the kinds of heightened emotional experiences that characterize live sport.

### 3.4 Visual cues, material presence, and the semiotics of green

Unlike the dimensions discussed above, visual encounters with sustainable infrastructure operate at both embodied and symbolic levels. Solar panels on stadium roofs, green walls and living roofs, and visible renewable technology in the surrounding landscape are material presences that spectators and athletes can see and interpret. Research on visible green design suggests that these elements influence environmental attitudes and behaviors among building occupants (26), functioning as what Latour (27) might call mediators that actively shape relationships between people and their environments.

However, a purely semiotic reading of these visual cues misses their embodied dimension. Solar panels do not merely symbolize sustainability; they alter light patterns, create shade, and change the visual texture of a building's silhouette against the sky. Green walls introduce organic forms, seasonal change, and living presence into built environments. These visual-material changes are experienced bodily—through the play of light on skin, the psychological effects of biophilic design, and the sense of being in a space that is alive and responsive rather than inert and industrial.

## 4 The embodied sustainable sports experience (ESSE) framework

**Figure 1:**
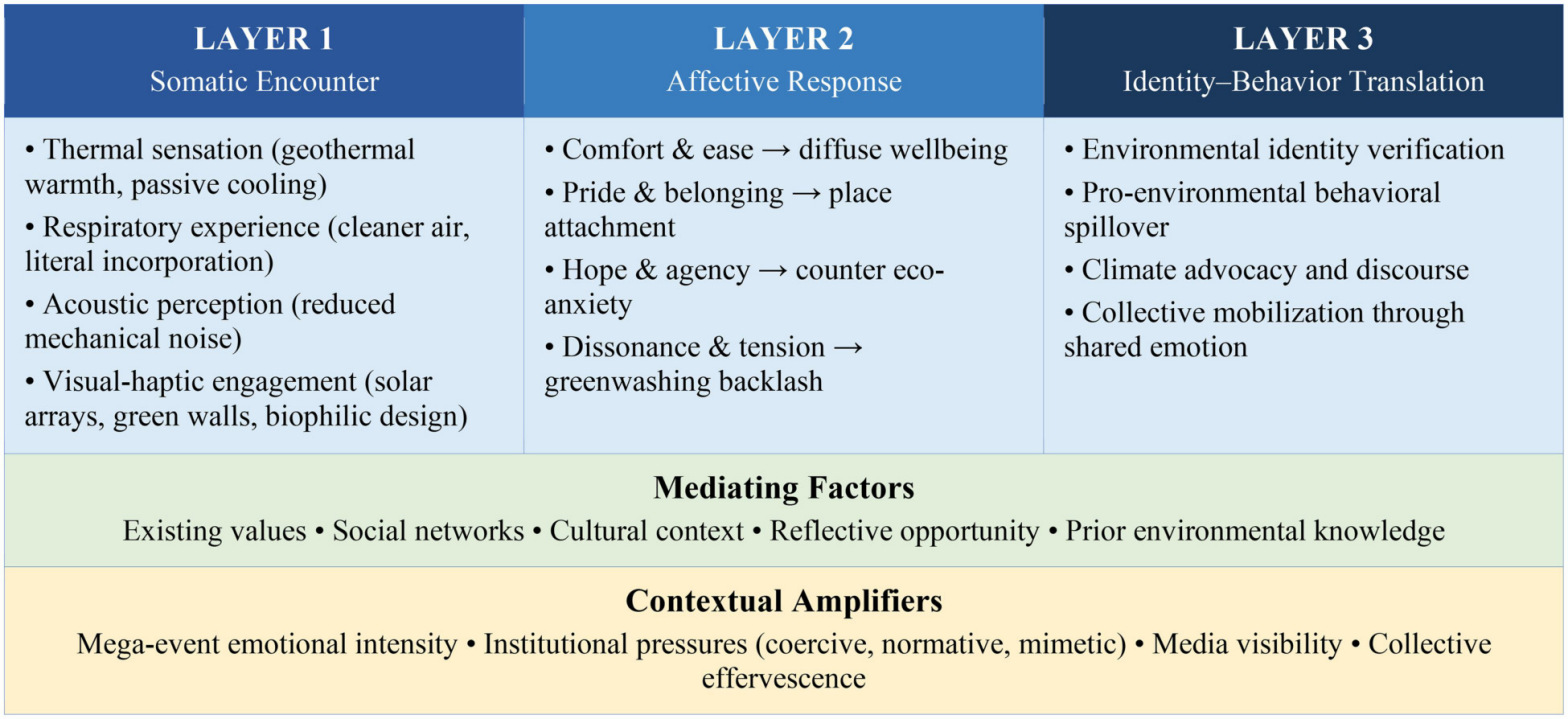
The embodied sustainable sports experience (ESSE) framework. Arrows between layers indicate directional influence; feedback loops connect Layer 3 outcomes back to Layer 1 through altered bodily dispositions and heightened attunement. The framework is conceptual and requires empirical validation.

Synthesizing the theoretical perspectives and embodied dimensions analyzed above, we now propose the Embodied Sustainable Sports Experience (ESSE) Framework. This framework maps the conceptual pathways through which sustainable energy infrastructure in sports venues may generate embodied and emotional experiences that promote engagement with climate action. The ESSE Framework operates across three interconnected layers: Somatic Encounter, Affective Response, and Identity-Behavior Translation. These layers are modulated by mediating factors (existing values, social networks, cultural context, reflective opportunity) and amplified by contextual conditions (mega-event intensity, institutional pressures, media visibility, collective effervescence). Figure 1 presents the framework visually.

### 4.1 Layer 1: somatic encounter

The first layer captures the pre-reflective, bodily engagement with sustainably designed environments analyzed in Section 3. Athletes and spectators encounter green infrastructure through thermal sensation, respiratory experience, acoustic perception, and visual-haptic engagement. These encounters are shaped both by the material properties of sustainable energy systems and by what Bourdieu (28) calls habitus—the embodied dispositions that individuals bring to environments. A seasoned athlete whose body is finely attuned to environmental conditions will register changes differently than a casual spectator, but both encounter the venue through their bodies. Crucially, many of these somatic impressions operate below the threshold of conscious awareness, shaping mood and orientation without deliberate reflection.

### 4.2 Layer 2: affective response

The second layer maps the emotional responses generated by somatic encounters. Drawing on the theoretical foundations outlined in Section 2, we identify four principal affective pathways:

Comfort and ease: When sustainable design produces environments that feel right—thermally balanced, well-ventilated, acoustically pleasant—bodies respond with a diffuse sense of wellbeing that colors the entire sporting experience. This is the affective register of Leder's (17) “eu-appearance,” and while it may not be consciously attributed to green infrastructure, it establishes a positive somatic baseline that predisposes occupants toward favorable evaluations of the venue and, by extension, of the sustainability choices it embodies.

Pride and belonging: When visible sustainability features are recognized and valued, they can generate feelings of pride in being associated with an environmentally responsible venue, strengthening place attachment (20) and group identity. For fans who already identify with a team or event, the venue's environmental credentials can become woven into the fabric of that identity—a source of collective distinction.

Hope and agency: Experiencing a tangible, functioning alternative to fossil fuel dependence can counter the helplessness associated with eco-anxiety (21). Standing in a stadium powered by solar energy, breathing air that is cleaner because the energy is cleaner, offers an embodied counternarrative to climate doom—evidence, registered by the body, that meaningful change is possible and already underway.

Dissonance and moral tension: When sustainability claims are perceived as superficial or contradicted by other aspects of the venue experience, the resulting emotional dissonance may produce frustration, cynicism, or active disengagement. If the signage says “green” but the air feels stale and the generators rumble underfoot, bodies know the difference, and the emotional fallout of this perceived hypocrisy can be corrosive.

### 4.3 Layer 3: identity-behavior translation

The third layer addresses how accumulated embodied-emotional experiences may translate into climate-conscious identities and behaviors. This translation is neither automatic nor universal; it is mediated by existing values, social networks, cultural contexts, and the degree to which individuals have opportunities to reflect on and discuss their experiences. However, the framework proposes that repeated, positive embodied encounters with sustainable sports environments can contribute to what Stets and Burke (29) call identity verification—the process through which environmental values become integrated into one's sense of self.

Mega-events may be particularly powerful catalysts for this identity work. Sarvinoz Atoevna et al. (6) demonstrated that major sporting events create institutional pressures—coercive, normative, and mimetic—that accelerate sustainability adoption in hospitality infrastructure. We extend this institutional analysis by arguing that mega-events also create emotional-experiential pressures: the heightened affective intensity of major competitions, combined with the visibility of sustainability initiatives and the force of collective effervescence, creates conditions under which embodied encounters with green infrastructure are amplified and shared. When forty thousand people simultaneously experience a sustainably powered venue, the emotional charge of that shared experience has the potential to create durable associations between sporting identity and environmental responsibility.

## 5 Implications for emerging sports tourism destinations

The ESSE Framework holds particular relevance for emerging sports tourism destinations, where the relationship between bodies, environments, and energy systems is being actively constructed rather than retrofitted. Central Asian countries, including Uzbekistan, Kazakhstan, and neighboring states, are increasingly hosting regional and international sporting events as part of broader strategies for cultural diplomacy and economic diversification, creating opportunities to embed sustainability into the embodied experience of sport from the very beginning (31–35).

Toyirova (7) demonstrated that sustained energy efficiency interventions in Uzbekistan achieved meaningful results in national infrastructure, with long-term decision-making frameworks outperforming short-term approaches. We argue that these findings carry an underexplored embodied dimension: the physical experience of living and working in more energy-efficient environments—warmer buildings in winter, cooler spaces in summer, cleaner air throughout the year—constitutes a form of bodily knowledge about sustainability that complements technical and economic data. When this embodied knowledge is concentrated in the emotionally charged environment of a sports venue, its potential to shape environmental attitudes is amplified in ways that policy documents and sustainability reports cannot replicate.

Emerging destinations hold a distinctive advantage: they can design sustainability into new facilities from inception rather than retrofitting existing structures. This means the embodied experience of green infrastructure can be intentional rather than incidental—architects, engineers, and event planners can deliberately create environments in which the felt qualities of sustainable energy (thermal comfort, air quality, acoustic character, visual identity) are optimized not only for operational performance but for the cultivation of environmental consciousness among the people who inhabit these spaces.

The cultural dimension merits attention as well. In many Central Asian contexts, the relationship between people and the natural environment is shaped by traditions of hospitality, seasonal awareness, and connection to landscape that differ from the individualistic, consumption-oriented frameworks dominating Western sustainability discourse. The ESSE Framework's emphasis on embodied and collective experience may resonate with cultural orientations that already value bodily attunement to environment and communal emotional experience, offering pathways to climate engagement that feel locally meaningful rather than imported.

## 6 Discussion

This conceptual analysis has argued that the embodied and emotional dimensions of sustainable energy transitions in sports tourism venues constitute a significant but undertheorized mechanism through which sport can promote climate action. By weaving together phenomenological perspectives on embodiment, the sociology of emotion, and the concept of affective atmospheres, we have sought to show that sustainability in sports infrastructure is not only a matter of engineering and economics but a matter of how people feel—in the fullest, most bodily sense of that word.

Several implications follow for both research and practice. First, future empirical work should attend closely to the sensory and emotional experiences of athletes and spectators in sustainably designed venues. Qualitative methodologies—sensory ethnography (30), phenomenological interviewing (2), and affect mapping—are better equipped to capture the embodied dimensions that survey instruments typically miss. Controlled comparisons between conventionally and sustainably powered venues, incorporating physiological measures (skin conductance, respiratory rate) alongside qualitative accounts, could provide empirical grounding for the ESSE Framework's conceptual claims.

Second, venue designers and event planners should consider the affective impact of sustainability features alongside their technical performance. A solar panel that is seen and understood may produce different atmospheric effects than one hidden from view; a geothermal system whose steady warmth is consciously experienced may foster different emotional responses than one that operates entirely behind the scenes. The implication is that sustainable design should be legible—not in the heavy-handed manner of greenwashing, but in ways that allow bodies to register the difference and minds to make meaning from it.

Third, the framework draws attention to the emotional dynamics of greenwashing as an embodied phenomenon. When sustainability claims are contradicted by bodily experience—when a venue branded as “green” feels stuffy, loud, and uncomfortable—the resulting emotional dissonance may be more damaging to environmental engagement than the absence of sustainability claims altogether. Authenticity, in the ESSE Framework, is not merely a marketing consideration but an embodied one: bodies are remarkably sensitive detectors of the gap between what is claimed and what is felt.

Fourth, the collective emotional dynamics of major sporting events—the shared elation, the communal tension, the collective sense of occasion—represent underutilized opportunities for weaving environmental consciousness into group identity. When thousands of people simultaneously experience a sustainably powered venue and share that experience through conversation, social media, and collective memory, the emotional charge of the shared moment can create durable associations between sporting community and environmental responsibility.

We acknowledge important limitations. The ESSE Framework is theoretical and requires empirical testing across diverse cultural, climatic, and institutional contexts. The pathways we propose between somatic encounter, affective response, and behavioral change are likely nonlinear and contingent on numerous mediating factors that we have identified but not fully specified. Our focus on the positive potential of embodied sustainable experiences should be balanced by systematic attention to the conditions under which such experiences fail to translate into lasting behavioral change, as well as the ways that bodily discomfort, emotional manipulation, and superficial sustainability claims can undermine genuine engagement.

## 7 Conclusion

Sport moves people—in every sense of the word. It moves bodies through space, moves emotions through their full range, and, at its best, moves people to care about the world beyond the playing field. This conceptual analysis has argued that the sustainable energy transitions reshaping sports infrastructure deserve attention not only as technical and economic phenomena but as transformations in the embodied, emotional texture of sporting life.

The ESSE Framework proposed here offers a conceptual vocabulary for understanding how the felt experience of sustainably powered venues—the warmth that comes from the earth rather than from burning gas, the air that is clean because the energy is clean, the visible presence of renewable technology woven into the sporting landscape—can reshape the way people relate to environmental issues. In doing so, it contributes to the growing recognition that climate action is not only a matter of policy and technology but of bodies, feelings, and the places where they come together.

For emerging sports tourism destinations, particularly in Central Asia and other transitional economies, this perspective offers both a challenge and an opportunity: the challenge of designing sustainable infrastructure that is not merely efficient but emotionally resonant, and the opportunity to create sporting environments where the experience of sustainability is embedded in the very fabric of what it feels like to be there. If we are to build sports venues that serve both athletic excellence and planetary wellbeing, we must attend not only to the energy they consume but to the feelings they produce.
